# Genome-wide analysis of chromatin architecture in *Oxytricha trifallax*: a single-celled eukaryote with 16,000 tiny chromosomes

**DOI:** 10.1186/1756-8935-6-S1-P4

**Published:** 2013-03-18

**Authors:** Leslie Y Beh, Laura F Landweber

**Affiliations:** 1 Department of Ecology and Evolutionary Biology, Princeton University NJ 08544, USA

## 

The pond-dwelling ciliate *Oxytricha trifallax* is a model system for the study of gene regulatory mechanisms. It possesses a heterochromatin-rich germline micronucleus, and a transcriptionally active somatic macronucleus (MAC). The MAC genome is highly fragmented, consisting of >16,000 unique “nanochromosomes”, with a mean size of 3.2kb. Consequently, only a limited number of nucleosomes can be accommodated in short nanochromosomes. Nanochromosome size does not vary in multiples of ~147bp corresponding to mono-nucleosome-sized DNA, suggesting that nucleosome depleted regions may be especially prevalent. This unusual genome architecture presents novel challenges for the regulation of gene expression. The efficacy and prevalence of chromatin-mediated gene regulation thus remain unclear in *Oxytricha*. In addition, the paucity of non-coding DNA suggests that promoters in *Oxytricha* are highly compact, and may be organized differently from other eukaryotes. Our study aims to uncover how the chromatin structure of promoters and coding regions is constrained by chromosome length in the vegetative *Oxytricha* MAC. To address this, we characterize the chromatin landscape of the MAC through genome-wide mapping of nucleosome positions and DNase I hypersensitive sites. By integrating these studies with existing RNA Polymerase II ChIP-seq and mRNA-seq data, we investigate how chromatin organization varies with nanochromosome length, gene number, and transcriptional output. These data will also be coupled with transcription start site annotations to define promoter architecture in the *Oxytricha* MAC, including the presence and position of regulatory motifs, the distribution of transcription start sites within a promoter, and local chromatin accessibility. Together, this integrated analysis will test the contribution of chromatin structure to gene expression regulation, and illuminate the variation of eukaryotic gene regulatory mechanisms.

